# Correlation Analysis of Lower Limb Contrast‐Enhanced Ultrasound Findings With Serum VEGF and CXCL‐12 Levels in a Rabbit Model of Diabetic Foot

**DOI:** 10.1155/bmri/5408596

**Published:** 2026-04-03

**Authors:** Yuzhong Wang, Tianlin Gao, Hong Zhou, Xudong Wang, Siyi Xie, Meiling Liu, Ming Li, Shuxing Cao, Huiyao Hao, Yongzhou Song, Zhe Lv

**Affiliations:** ^1^ Department of Orthopedics, The Second Hospital of Hebei Medical University, Shijiazhuang, Hebei, China, hebmu.edu.cn; ^2^ Hebei Medical University, Shijiazhuang, Hebei, China, hebmu.edu.cn; ^3^ Department of Cardiac Ultrasound, Luquan Branch, The Second Hospital of Hebei Medical University, Shijiazhuang, Hebei, China, hebmu.edu.cn; ^4^ Department of Medical Equipment, The Second Hospital of Hebei Medical University, Shijiazhuang, Hebei, China, hebmu.edu.cn; ^5^ Department of Endocrinology, The Second Hospital of Hebei Medical University, Shijiazhuang, Hebei, China, hebmu.edu.cn; ^6^ Department of Otolaryngology, The Second Hospital of Hebei Medical University, Shijiazhuang, Hebei, China, hebmu.edu.cn

**Keywords:** contrast-enhanced ultrasound, CXCL-12, diabetic foot, microcirculation, New Zealand white rabbit, VEGF

## Abstract

**Objective:**

We are aimed at analyzing the correlation between contrast‐enhanced ultrasound (CEUS) quantitative perfusion parameters and serum levels of vascular endothelial growth factor (VEGF) and C‐X‐C motif chemokine ligand 12 (CXCL‐12) in a rabbit model of diabetic foot, and exploring the value of CEUS in evaluating microcirculatory lesions of diabetic foot.

**Methods:**

Ten New Zealand white rabbits were intravenously injected with alloxan to induce diabetes (fasting blood glucose > 16 mmol/L, accompanied by symptoms of polydipsia, polyphagia, and polyuria). Diabetic foot ulcer (DFU) was then induced by magnetic disk compression. Serum VEGF and CXCL‐12 levels were measured by enzyme‐linked immunosorbent assay (ELISA) at three stages (normal stage, diabetes mellitus [DM] stage, and DFU stage). CEUS was performed on the gastrocnemius muscle to quantify three perfusion‐related parameters derived from time‐intensity curves: time to peak (TTP), peak intensity (PI), and area under the curve (AUC).

**Results:**

Serum VEGF and CXCL‐12 levels in diabetic rabbits were significantly higher than those in normal rabbits (*p* < 0.01); in DFU rabbits, these levels remained elevated but were slightly lower than those in diabetic rabbits (*p* > 0.1). CEUS‐derived perfusion parameters (TTP, PI, and AUC) showed an increasing trend across disease stages, with the highest values observed in the DFU group. All three parameters were significantly positively correlated with serum VEGF and CXCL‐12 levels (*r* = 0.509–0.649, *p* < 0.01).

**Conclusion:**

Serum VEGF and CXCL‐12 levels increase in diabetes but slightly decrease when DFU develops, indicating microcirculatory decompensation. CEUS‐derived perfusion parameters, especially TTP, can sensitively reflect microcirculatory impairment and are positively correlated with angiogenic factor levels. This study suggests that CEUS provides a novel noninvasive tool for evaluating microcirculatory lesions in diabetic foot, with potential clinical translational value.

## 1. Introduction

Diabetes mellitus (DM) is a multifactorial chronic metabolic disorder characterized by elevated blood glucose and insulin resistance [1]. In recent years, the global prevalence of DM has increased sharply. Due to the long course of the disease and poor control, up to 25% of patients develop diabetic foot ulcer (DFU) [2]. DFU is directly associated with difficult wound healing and may lead to the serious consequence of lower limb amputation. The formation of new blood vessels in the affected limb is a key stage in the wound healing process, as this process ensures the delivery of oxygen and nutrients to the wound site, promoting effective healing [3]. Therefore, angiogenesis plays a crucial role in the mechanism of wound healing in diabetic foot.

In the process of vascular lesion development, both vascular endothelial growth factor (VEGF) and C‐X‐C motif chemokine ligand 12 (CXCL‐12) can promote angiogenesis. VEGF is considered a key stimulator of angiogenesis, exerting positive effects on angiogenesis in aspects such as vasodilation and permeability, degradation of the vascular extracellular matrix (ECM), proliferation and migration of endothelial cells (EC), lumen formation, and vascular stabilization [4–6]. CXCL‐12, also known as stromal cell–derived factor‐1 (SDF‐1), is a small‐molecule cytokine with angiogenic activity and a significant relationship with insulin resistance [7, 8]. In summary, we believe that VEGF and CXCL‐12 have biological correlations with angiogenesis and microcirculatory permeability [9–11].

According to the 2016 American Heart Association (AHA)/American College of Cardiology (ACC) Guidelines for the Management of Patients with Lower Extremity Peripheral Arterial Disease (PAD), duplex ultrasound (DUS), computed tomography angiography (CTA), digital subtraction angiography (DSA), and magnetic resonance angiography (MRA) are all reasonable diagnostic imaging methods for evaluating the anatomical characteristics of PAD [12]. Each method has its advantages and limitations (see Table 1), and x‐ray angiography/DSA remains the gold standard for peripheral arterial imaging. However, due to the invasive nature of DSA and the risk of renal injury from contrast agents, caution is required when using DSA in diabetic patients with microcirculatory complications. Therefore, there is currently a lack of effective dynamic and noninvasive methods for evaluating extremity microcirculation in DFU patients. Contrast‐enhanced ultrasound (CEUS) is a blood perfusion imaging technique that can real‐time and quantitatively evaluate tissue microcirculatory perfusion [13], and has been widely used in the evaluation of lesions in the heart, liver, kidneys, and other organs [14, 15]. However, few studies have reported the use of CEUS to evaluate microcirculatory perfusion in diabetic foot. In this study, a rabbit model of diabetic foot was established. Due to the superficial location, clear anatomical structure, and extensive microvascular network of the gastrocnemius muscle, the gastrocnemius muscle was selected as the research object during CEUS [16, 17]. The aim of this study was to investigate the changes in VEGF and CXCL‐12 before and after modeling, as well as the quantitative changes in CEUS of the rabbit lower limbs, and analyze the correlation between microcirculatory changes in diabetic foot and the levels of VEGF and CXCL‐12 in the body, in order to provide a basis for clinical evaluation of peripheral arterial lesion.

**Table 1:** Comparison of vascular lesion assessment methods for lower extremity peripheral arterial disease [12].

Assessment method	DSA	DUS	CTA	MRA	CEUS
Resolution	Gold standard	Operator‐dependent	High resolution.	High resolution	Operator‐dependent
Invasiveness	Invasive (requires puncture/iodinated contrast agent)	Noninvasive (no exogenous contrast agent required)	Noninvasive.	Noninvasive	Invasive (requires puncture/microbubble contrast agent)
Imaging dimension	Two‐dimensional	Two‐dimensional	Three‐dimensional image reconstruction.	Three‐dimensional image reconstruction	Two‐dimensional
Special advantages	Real‐time dynamic imaging	Provides real‐time hemodynamic information	Fast scanning (< 5 min).	Excellent contrast for soft tissue components of plaques; evaluation of infrapopliteal artery lesions is not affected by blood flow; and can provide hemodynamic/blood flow information	Provides real‐time hemodynamic information, no ionizing radiation, and no kidney injury risk
Main limitations	Limited assessment of complete occlusion; has ionizing radiation; limited assessment of vascular wall/plaque burden; invasive; contrast agent has a risk of kidney injury, and should be used with caution in DFU patients	Limited field of view and limited assessment of severely calcified blood vessels	Has ionizing radiation; difficult to master the contrast agent injection time; limited assessment of severely calcified blood vessels; and limited assessment of infrapopliteal artery lesions.	May require gadolinium contrast agent in some cases: cannot achieve the same high‐resolution data acquisition time as CTA; patients may have contraindications; and not suitable for patients with claustrophobia.	Limited field of view

Abbreviations: CEUS, contrast‐enhanced ultrasound; CTA, computed tomography angiography; DFU, diabetic foot ulcer; DSA, digital subtraction angiography; DUS, duplex ultrasound; MRA, magnetic resonance angiography.

## 2. Materials and Methods

### 2.1. Animal Experimental Materials

#### 2.1.1. Ethical Approval

The animal experimental protocol was reviewed and approved by the Ethics Committee of The Second Hospital of Hebei Medical University (Ethics Approval No. 2022‐R028). All procedures were conducted in accordance with institutional guidelines for the care and use of laboratory animals.

#### 2.1.2. Experimental Animals

Male New Zealand white rabbits (conventional grade, Certificate No. 41098325113369960) were used for modeling. The animals were purchased from Henan Suke Beisi Biotechnology Co., Ltd. (SCXK (Yu) 2025‐0005). All experimental rabbits were 3 months old, with a body weight of approximately 2.5–3.0 kg (*N* = 10). They were individually housed by designated personnel under controlled conditions: a temperature of 21°C, a 12‐h light/dark cycle, and 60% humidity. After 1 week of acclimatization, they were confirmed to be free of other diseases.

#### 2.1.3. Main Reagents

• Zoletil 50 (Virbac, France)
• Alloxan (Hefei Bomei Biotechnology Co., Ltd., China)
• Gensulin N (Jilin Minshengyuan Pharmacy Co., Ltd., China)
• Lidocaine injection (Sinopharm Rongsheng Pharmaceutical Co., Ltd., China)
• Sulfur hexafluoride microbubbles for injection (Bracco Suisse SA, Switzerland)
• Rabbit VEGF ELISA Kit, Rabbit CXCL‐12 ELISA Kit (Jiangsu Jingmei Biotechnology Co., Ltd., China)
• RIPA lysis buffer (containing PMSF), protease inhibitor, phosphatase inhibitor, BCA protein concentration assay kit (containing BSA standard), 30% acrylamide, 1 × Tris − HCl buffer (pH = 6.8), 1.5 × Tris − HCl buffer (pH = 8.8), 10% SDS (Solarbio, China).
• TBS buffer (Boster Biological Technology, China)

### 2.2. Animal Experimental Methods

**Figure 1:**
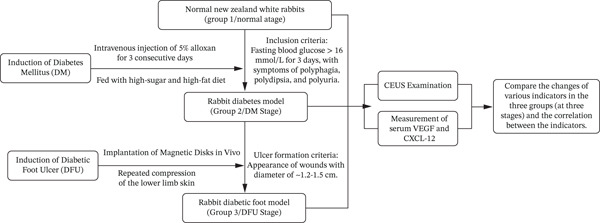
Flowchart of the experimental design. *Note:* DM, diabetes mellitus; DFU, diabetic foot ulcer; CEUS, contrast‐enhanced ultrasound; VEGF, vascular endothelial growth factor; CXCL‐12, C‐X‐C motif chemokine ligand 12.

The overall experimental design, including the procedures for model establishment, grouping, and data collection, is summarized in Figure 1.

#### 2.2.1. Establishment of Experimental Animal Models and Grouping

##### 2.2.1.1. Establishment of DM Model.

After the experimental animals were enrolled, they were first fed with regular feed for 1 week of acclimatization, followed by 1 week of high‐fat and high‐sugar feed. If no obvious adverse reactions were observed in the animals, 5% alloxan solution at a dose of 100 mg/kg was injected intravenously via the marginal ear vein for 3 consecutive days to induce diabetes [18]. During this process, the blood glucose changes of the experimental animals were closely monitored. If either of the following criteria were met, the intravenous injection of alloxan solution was stopped: fasting blood glucose > 16.0 mmol/L for 3 consecutive days, or a single random blood glucose > 20.0 mmol/L. Insulin (Gensulin N) was subcutaneously injected daily, with the dose adjusted in increments of 1 U based on the measured blood glucose levels, starting from an initial dose of 1 U. When the experimental animals showed symptoms of polydipsia, polyphagia, and polyuria, and the fasting blood glucose was controlled between 16 and 25 mmol/L, the DM model was considered successfully established [19].

##### 2.2.1.2. Establishment of Diabetic Foot Ulcer Model.

**Figure 2:**
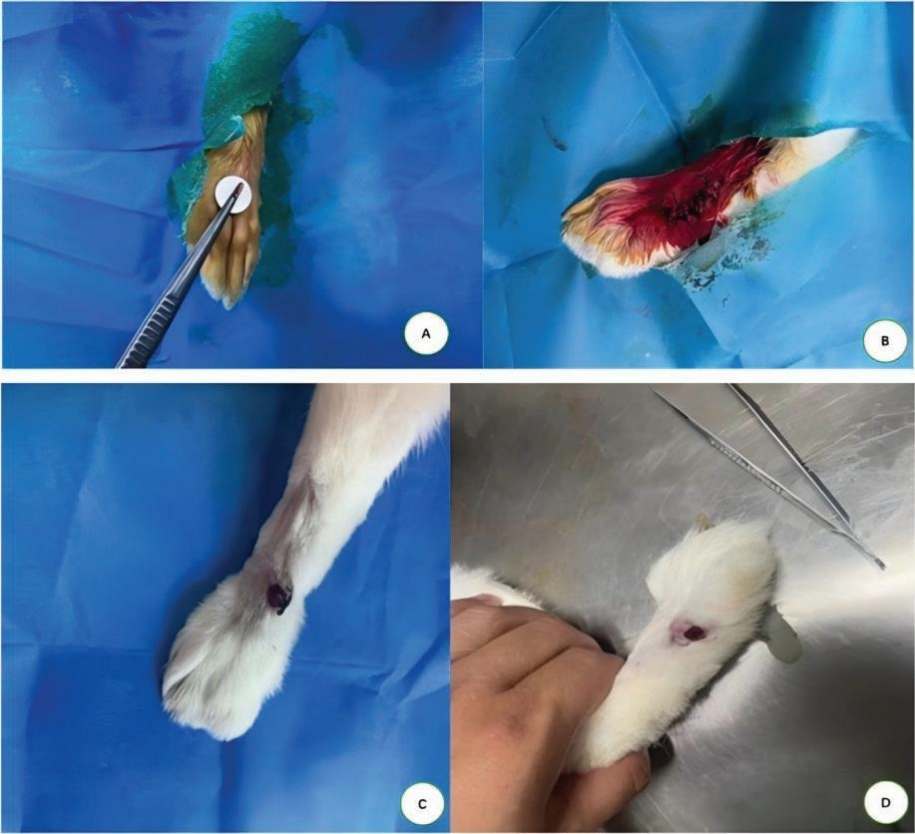
Establishment of a diabetic foot model in New Zealand white rabbits. (A, B) Surgical implantation of magnetic disks. (C, D) Successful induction of the diabetic foot ulcer.

The rabbits were anesthetized by intravenous injection of Zoletil 50 (0.1 mL/kg) via the marginal ear vein. Following anesthesia, the hind feet were depilated, and magnetic disks were surgically implanted [20]. After the incisions healed, external magnetic disks were placed over the skin, where they magnetically attracted and pressed against the internally implanted disks to compress the local tissue. Ulcer induction was conducted using a cyclic compression protocol: 2 h of pressing, 30 min of relaxation, and another 2 h of pressing. This cycle was repeated daily until an ulcer with a diameter of approximately 1.2–1.5 cm was induced (see Figure 2). During the process, one rabbit experienced magnetic disk detachment, which was addressed by replacing the rabbit with another one of the same type.

##### 2.2.1.3. Study Design.

This was a within‐subject, longitudinal study. Each animal served as its own control, with all outcome measures assessed at three sequential time points: normal stage, DM stage, and DFU stage.

##### 2.2.1.4. Sample Size.

The sample size (*n* = 10) was determined based on previous similar studies using rabbit models of diabetic foot. No formal a priori power calculation was performed due to the exploratory nature of this study.

##### 2.2.1.5. Inclusion and Exclusion Criteria.

Animals were included if they successfully met the DM induction criteria (fasting blood glucose > 16 mmol/L for 3 consecutive days). Exclusion criteria were established a priori and included failure to meet DM criteria after alloxan injection, magnetic disk detachment that could not be reimplanted, or development of severe infection or other complications requiring early euthanasia. One rabbit was excluded due to magnetic disk detachment and was replaced with another animal of the same age and weight.

##### 2.2.1.6. Randomization.

No randomization was applied, as this was a within‐subject design. The left hind limb was consistently used for CEUS examination in all animals to ensure consistency.

##### 2.2.1.7. Blinding.

The physician performing CEUS examinations was not blinded to the experimental stages, as the visible ulcer in the DFU stage made blinding impossible. However, quantitative analysis of perfusion parameters was performed using automated software to minimize bias. Serum ELISA measurements were performed by a technician who was blinded to the experimental stages.

##### 2.2.1.8. Animal Monitoring and Humane Endpoints.

Animals were monitored daily for signs of pain, distress, or illness, including changes in body weight, behavior (e.g., grooming, feeding, and mobility), and wound condition (e.g., signs of infection like redness, swelling, or discharge). Humane endpoints were established a priori. An animal would be humanely euthanized if it exhibited any of the following: rapid weight loss exceeding 20% of baseline body weight, severe infection not controlled by debridement, signs of extreme pain or distress not alleviated by analgesics, or inability to eat or drink. No animal met these endpoints during the study.

##### 2.2.1.9. Euthanasia.

At the conclusion of the experiment, all rabbits were humanely euthanized by intravenous injection of an overdose of Zoletil 50 (0.4 mL/kg) via the marginal ear vein. Death was confirmed by respiratory arrest and absence of cardiac activity. The procedure was performed in accordance with the AVMA Guidelines for the Euthanasia of Animals.

### 2.3. Measurement of Serum VEGF and CXCL‐12

At the normal, DM, and DFU stages, blood samples were collected from the marginal ear vein of fasting rabbits. Serum levels of VEGF and CXCL‐12 were measured using enzyme‐linked immunosorbent assay (ELISA). The absorbance of the standard solutions and serum samples at 450 nm was measured with a microplate reader. A standard regression curve was plotted to calculate the serum concentrations of VEGF and CXCL‐12. The levels of VEGF and CXCL‐12 across different stages (representing different experimental groups) were recorded and compared to analyze the dynamic changes of these angiogenic factors during diabetic progression and their association with microcirculatory dysfunction.

#### 2.3.1. CEUS Examination

A Philips EPIQ7c ultrasonic diagnostic instrument with an L12‐3 linear array probe was used. Before CEUS, conventional ultrasound scanning of the gastrocnemius muscle was performed first. Color Doppler was used to determine the location of arteries and veins in the skeletal muscle as positioning markers. CEUS examination was conducted again before modeling, after DM modeling, and after diabetic foot model establishment.

##### 2.3.1.1. CEUS Procedure.

The contrast agent SonoVue (Bracco) was mixed with 5 mL of normal saline and shaken to form a suspension for later use. A venous access was established via the ear margin vein. Each time, Zoletil 50 solution was intravenously injected at a dose of 0.1 mL/kg. After successful anesthesia, the rabbit was positioned, and the left lower limb was fully exposed and relaxed, with hair removed using a clipper. An L12‐3 linear array probe was used, and the musculoskeletal examination mode was adjusted. 2D ultrasound scanning was performed on the thickest part of the left gastrocnemius muscle of the experimental rabbit to determine the location of arteries and veins in the skeletal muscle. The system was then switched to CEUS mode, and the mechanical index (MI) was set to 0.07. The contrast agent was fully shaken and mixed, and then injected as a bolus via the ear margin vein at a dose of 0.2 mL/kg, followed by a rapid injection of 5 mL of normal saline to flush the pipeline. Immediately after the injection of the contrast agent, a timer was started and images were collected to observe the microcirculatory perfusion in the target area in real time and continuously [21]. During the contrast process of all animals, all parameters remained unchanged. After the operation, the experimental data were stored for further analysis.

##### 2.3.1.2. Image Analysis.

On the contrast images, two regions of interest (ROIs) with a diameter of approximately 5 mm were selected, avoiding bone, connective tissue, and large blood vessels. The time‐intensity curve (TIC) was generated using the built‐in ultrasound software, and the following perfusion parameters were obtained for each ROI: peak intensity (PI), area under the curve (AUC), and time to peak (TTP). The CEUS examination and analysis were performed by a physician with 10 years of experience, assisted by a technician.

#### 2.3.2. Statistical Methods

SPSS 25 software was used for statistical analysis. First, a normality test was performed on the data. Since the data did not conform to a normal distribution, the data were expressed as median (interquartile range) M (P25, P75). Friedman test was used to analyze the intergroup differences in CEUS parameters and serum VEGF and CXCL‐12 levels, with a significance level of *p* < 0.05. Spearman rank correlation test was used to analyze the correlation between CEUS indicators and serum VEGF and CXCL‐12 levels, with *p* < 0.05 considered statistically significant.

## 3. Results

### 3.1. Changes in Gastrocnemius CEUS Parameters During the Normal Stage, DM Stage, and DFU Stage

**Figure 3:**
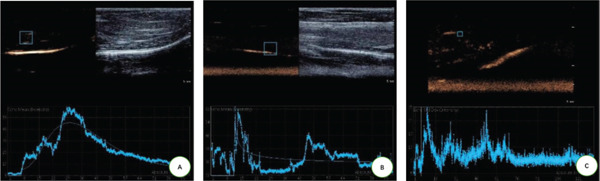
CEUS analysis at different modeling stages. (A) Normal stage: The time‐intensity curve (TIC) is flat, showing a slow–rise and slow–fall pattern, reflecting balanced microcirculatory blood flow (consistent inflow and outflow). (B) DM stage: The TIC presents a fast‐in and fast‐out pattern with a secondary perfusion peak, suggesting increased early microvascular permeability and residual blood flow compensation. (C) DFU stage: The TIC shows a fast‐in and fast‐out pattern without a secondary perfusion peak, accompanied by tailing, indicating microvascular occlusion and venous return disorder.

There were statistically significant differences in TTP, PI, and AUC among the three stages (*p* < 0.05). Compared with the normal stage, TTP, PI, and AUC in the DM stage and DFU stage were all increased (*p* < 0.05); compared with the DM stage, TTP, PI, and AUC in the DFU stage were increased (Table 2 and Figure 3).

**Table 2:** Changes in various indicators at different modeling stages (normal stage, DM stage, and DFU stage) (M [P25, P75]).

Indicator	Normal stage	DM stage	DFU stage	*χ* ^2^	*p*
TTP	11.29 (10.95, 11.91)	28.93 (28.79, 29.87)	130.41 (128.76, 132.42)	20.00	< 0.001
PI	25.74 (25.18, 26.29)	39.40 (38.87, 40.39)	41.72 (40.30, 42.13)	19.54	< 0.001
AUC	361.58 (356.67, 364.49)	1500.55 (1488.93, 1501.33)	2146.57 (2124.98, 2199.70)	20.00	< 0.001
VEGF	98.64 (91.27, 104.86)	163.27 (130.18, 177.53)	138.49 (130.45, 144.38)	15.80	< 0.001
CXCL‐12	5.97 (5.81, 6.17)	10.70 (10.29, 11.71)	9.92 (9.31, 10.76)	15.20	0.001

*Note:* Significant differences were observed in the changes of contrast‐enhanced ultrasound (CEUS) indicators (including time to peak [TTP], perfusion intensity [PI], and area under the curve [AUC]), and growth factor indicators (vascular endothelial growth factor [VEGF] and C‐X‐C motif chemokine ligand 12 [CXCL‐12]) across different stages of diabetic foot ulcer (DFU) development (*p* < 0.001).

### 3.2. Changes in VEGF and CXCL‐12 Levels During the Normal Stage, DM Stage, and DFU Stage

There were statistically significant differences in VEGF and CXCL‐12 among the three stages (*p* < 0.05). Compared with the normal stage, VEGF and CXCL‐12 in the DM stage and DFU stage were all increased (*p* < 0.05); compared with the DM stage, the changes in VEGF and CXCL‐12 in the DFU stage were not statistically significant (*p* > 0.05) (Table 2 and Figure 4).

Figure 4Changes in various indicators at different modeling stages (violin plot). (a–c) Changes in contrast‐enhanced ultrasound (CEUS) parameters (time to peak [TTP], perfusion intensity [PI], area under the curve [AUC]) across stages. (d, e) Changes in vascular endothelial growth factor (VEGF) and C‐X‐C motif chemokine ligand 12 (CXCL‐12) levels across stages. *Note:* DM, diabetes mellitus; DFU, diabetic foot ulcer; CEUS, contrast‐enhanced ultrasound; TTP, time to peak; PI, perfusion intensity; AUC, area under the curve; VEGF, vascular endothelial growth factor; CXCL‐12, C‐X‐C motif chemokine ligand 12.

**Figure:**
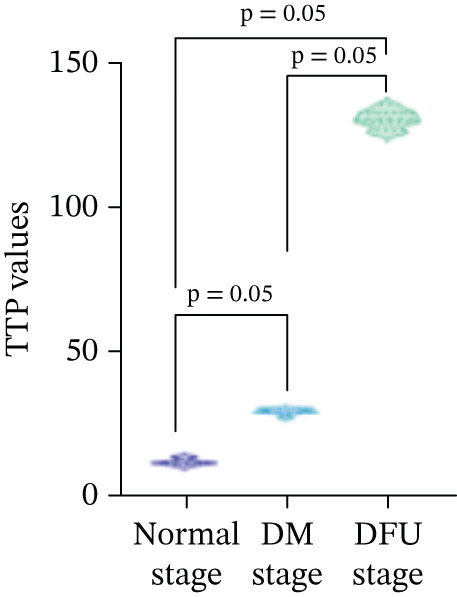
(a)

**Figure:**
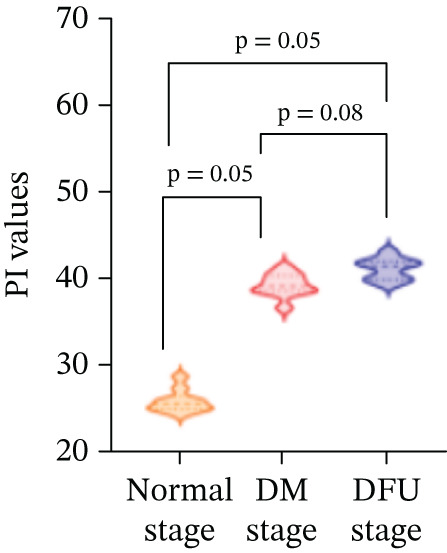
(b)

**Figure:**
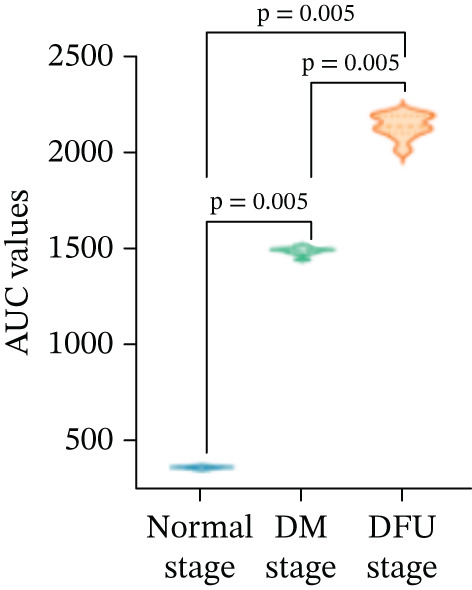
(c)

**Figure:**
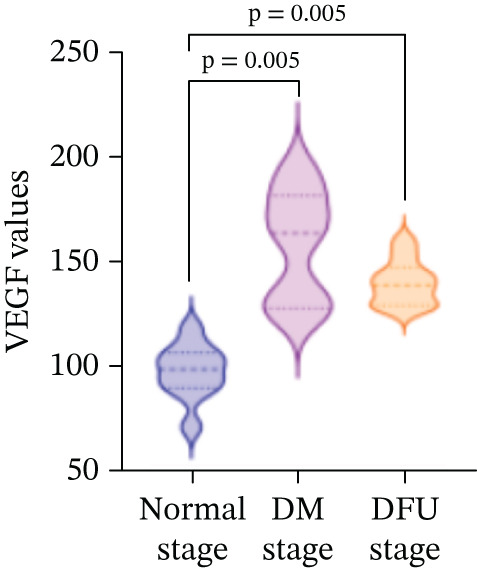
(d)

**Figure:**
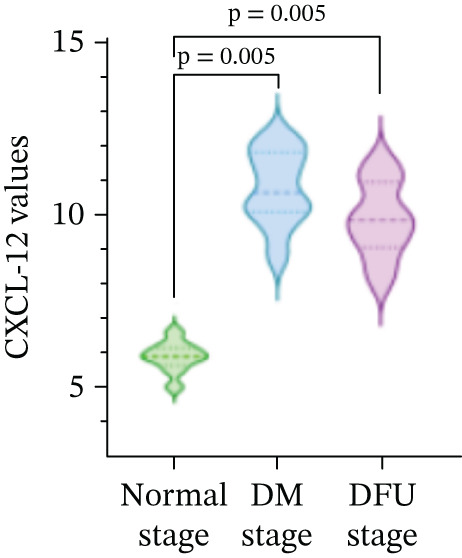
(e)

### 3.3. Correlation Between CEUS Parameters and VEGF, CXCL‐12 During the Normal Stage, DM Stage, and DFU Stage

All data before and after modeling in each group were integrated (*N* = 30) to analyze the correlation between CEUS parameters and growth factor levels at each stage. The three CEUS parameters (TTP, PI, and AUC) were significantly positively correlated with VEGF and CXCL‐12 (*r* = 0.509–0.649, *p* < 0.01). Among them, TTP showed the strongest correlation (VEGF: *r* = 0.649; CXCL‐12: *r* = 0.611) (Table 3).

**Table 3:** Correlation analysis between various indicators.

Parameter	TTP		PI		AUC	
	*r*	*p*	*r*	*p*	*r*	*p*
VEGF	0.649 ^∗∗^	< 0.001	0.584 ^∗∗^	0.001	0.606 ^∗∗^	< 0.001
CXCL‐12	0.611 ^∗∗^	< 0.001	0.536 ^∗∗^	0.002	0.509 ^∗∗^	0.004

*Note:* Three contrast‐enhanced ultrasound (CEUS) parameters, namely time to peak (TTP), perfusion intensity (PI), and area under the curve (AUC), all showed a significantly positive correlation with vascular endothelial growth factor (VEGF) and C‐X‐C motif chemokine ligand 12 (CXCL‐12) (*r* = 0.509–0.649, *p* < 0.01). Among these parameters, TTP exhibited the strongest correlation (VEGF: *r* = 0.649; CXCL‐12: *r* = 0.611).

^∗∗^ *p* < 0.01.

## 4. Discussion

Since skeletal muscle plays an important role in glucose metabolism, the occurrence of microcirculatory disorders will seriously affect the control of glucose metabolism in diabetic patients, thereby aggravating glucose metabolism disorders [22]. Currently, there are few dynamic and noninvasive methods for evaluating skeletal muscle microcirculation. Dynamic contrast‐enhanced magnetic resonance imaging and special sequences of magnetic resonance imaging can meet the requirements of dynamic evaluation and have good repeatability. They can be used to measure the maximum PI of the contrast agent and the TTP, enabling quantitative analysis of muscle microcirculation for the assessment of skeletal muscle perfusion [23, 24]. However, this method has not been widely used in clinical practice yet. CEUS is a relatively noninvasive examination method that can real‐time evaluate the microcirculatory perfusion and blood flow distribution of the limbs. The blood flow velocity in blood vessels can be quantitatively expressed through the process of contrast agent perfusion and clearance, and it has been widely used in the detection and quantification of perfusion changes in muscle tissue and visceral tissue [25, 26]. In this study, CEUS quantitative software was used to record the TIC during the contrast process. Among them, the blood flow volume during the microcirculatory filling phase can be reflected by PI and AUC; whereas TTP reflects the time efficiency of the contrast agent perfusing in the tissue and reaching the maximum enhancement, thereby reflecting the filling speed of the tissue microcirculation. The results showed that TTP, PI, and AUC increased sequentially in the normal stage, DM stage, and DFU stage, with statistically significant differences. However, TTP showed the most significant difference during the establishment of the diabetic foot model, and PI and AUC showed the most significant changes during the establishment of the DM model. The distinct patterns of parameter change across stages likely reflect a shifting pathophysiological dominance during the progression of diabetic microangiopathy in our acute model. Critically, this acute model may predominantly capture the early, dynamic phase of diabetic microvascular dysfunction, which is characterized by hemodynamic dysregulation and compensatory responses, rather than the end‐stage structural rarefaction seen in chronic disease. In the DM stage, the significant increases in PI and AUC may primarily indicate early functional disturbances in the local skeletal muscle microvasculature [27]. These include increased microvascular permeability and impaired venular outflow, leading to local pooling and delayed clearance of the contrast agent. This represents a state of hemodynamic dysregulation and functional decompensation within the muscle bed itself, effectively a pathological “hyper‐perfusion” state due to leakage and stasis, not adequate nutrient delivery [28].

The transition to the DFU stage is marked by the most dramatic prolongation of TTP. This suggests the superimposition of a new, dominant hemodynamic insult: significant inflow obstruction. Although progressive local capillary stenosis contributes [29], the extreme TTP prolongation strongly implies a major increase in upstream resistance [30, 31], likely originating from the severely compromised distal microcirculation of the foot induced by the ulcer model, which aligns with the established concept of distal microvascular occlusion in diabetic foot pathology [32]. Thus, the DFU stage evolves into a state of severe perfusion failure characterized by the synergy of persistent local microvascular dysfunction and acute distal inflow obstruction.

This pathophysiological progression—from early dysregulation to late obstruction—provides a coherent framework to interpret the CEUS findings in our DM model. The observed “hyper‐perfusion” pattern (elevated PI/AUC) is interpreted as a specific form of early microcirculatory failure. In diabetic neuropathy, loss of sympathetic tone and pathological opening of arteriovenous shunts can lead to a state of “non‐nutritive hyperemia,” where total blood flow increases at the expense of nutritive capillary perfusion [33]. This classic hemodynamic derangement aligns with our hypothesis of early functional decompensation and directly explains the elevated PI and AUC due to increased but maldistributed flow.

The pattern of elevated PI and AUC observed in our DM animal model, along with the subsequent TTP prolongation in DFU, provides a dynamic depiction of microvascular evolution. Our acutely induced model, focusing on proximal skeletal muscle (gastrocnemius), captures the early neurogenic and hemodynamic phase of diabetic microangiopathy, characterized by dysregulated flow and shunting [33, 34]. This phase may represent a critical transitional window in the natural history of the disease. Clinical studies often assess distal beds in patients with long‐standing diabetes, where structural changes and fixed hypoperfusion are predominant [35]. Therefore, our model offers a complementary perspective, illuminating the earlier functional disturbances that precede the well‐documented late‐stage structural ablation. The dynamic evolution of CEUS parameters observed here underscores their potential utility as biomarkers for staging disease progression and identifying early intervention points, which warrants further translational investigation.

The serum dynamics of VEGF and CXCL‐12 observed in this study—significantly elevated in DM and slightly declined yet still elevated in early DFU—can be interpreted within a dynamic “compensation‐to‐decompensation” framework of diabetic microangiopathy.

### 4.1. Compensatory Phase (Elevated Factors)

In the early DM stage, the organism enters a systemic stress state characterized by metabolic disturbances, oxidative stress, and inflammation. This stress response activates key adaptive pathways. Specifically, the PI3K/AKT signaling pathway, a central regulator of cellular adaptation to stressors like hyperglycemia and hypoxia, is activated and directly upregulates VEGF expression as a compensatory proangiogenic response. Concurrently, oxidative stress–mediated transcription factors drive the elevation of CXCL‐12, a key chemokine for recruiting repair cells. This systemic upregulation of angiogenic factors represents an attempt to maintain vascular homeostasis and preemptively counteract injury, a phenomenon analogous to stem cell–mediated tissue repair mechanisms observed in other stress states like infarction [36]. At this stage, microcirculatory alterations may be subtle, as potentially reflected in early CEUS evaluations showing minor changes, indicating preserved functional reserve.

### 4.2. Transition to Decompensation (Factor Decline)

The subsequent slight decline in these factors during early DFU signals the onset of decompensation. Critically, this systemic elevation, even if slightly diminished, is not indicative of successful repair but rather of a failing compensatory state. This is strongly supported by clinical evidence: Elevated serum VEGF is an independent risk factor for nonhealing/recurrent DFU, and CXCL‐12 remains high in active, neuropathy‐dominant ulcers but collapses in late vascular insufficiency [37, 38]. Persistent stress leads to increased apoptosis of endothelial and progenitor cells, depleting the cellular source of VEGF and CXCL‐12 [39]. This aligns with the significant decline in limb microcirculatory passage capacity observed via CEUS in late‐stage diabetes in our study. The progression from subtle to severe microcirculatory dysfunction underscores a critical failure of endogenous repair mechanisms, creating a therapeutic imperative. This context is highly relevant to advanced material‐based strategies aimed at stabilizing the wound microenvironment and enhancing recovery. For instance, multifunctional nanocomposites have been designed to improve wound healing under stress by modulating the local milieu [40], and microneedle systems enhance drug delivery and tissue remodeling in late‐stage chronic wounds [41]. These approaches parallel concepts where nanoparticles or aptamers are used to precisely modulate pathological pathways (e.g., PI3K) in other diseases [42], highlighting a potential strategy for targeted intervention in diabetic microcirculatory failure.

Therefore, our data, interpreted through this mechanistic and translational framework, illuminate a continuum from stress adaptation to microcirculatory failure. This progression not only explains our biomarker and CEUS findings but also identifies a critical window for intervention, where strategies inspired by regenerative medicine and advanced biomaterials could help restore microcirculatory function before irreversible damage occurs.

### 4.3. Limitations

This study has several limitations. First, the small sample size may have introduced experimental errors; thus, our findings require further validation in future large‐scale prospective studies utilizing larger animal models. Second, there is currently no gold standard for the quantitative measurement of limb microcirculation, and our contrast perfusion results lack comparative controls. Therefore, future large‐sample studies should incorporate microcirculatory pathological examinations to further investigate the mechanisms underlying microcirculatory changes during diabetic foot formation.

## 5. Conclusion

DFU is a complex disease process characterized by heterogeneous lesions and intricate pathological mechanisms. Vascular wall pathology and complex hemodynamics are key features of this disease; however, current imaging techniques struggle to clearly evaluate patient microcirculatory characteristics. As an imaging tool for the dynamic assessment of microcirculation, CEUS demonstrates that serum levels of VEGF and CXCL‐12 positively correlate with CEUS‐evaluated parameters (PI, AUC, and TTP), which may be associated with the degree of microcirculatory impairment during disease progression. CEUS provides a new approach for the future dynamic evaluation of microcirculation in DFU and may demonstrate significant advantages.

## Author Contributions

**Yuzhong Wang:** conceptualization, methodology, investigation, writing – original draft. **Tianlin Gao:** writing – review and editing, visualization. **Hong Zhou:** data curation, formal analysis. **Xudong Wang:** resources, investigation. **Siyi Xie:** data curation, visualization. **Meiling Liu:** data curation, formal analysis. **Ming Li:** writing – review and editing. **Shuxing Cao:** writing – review and editing. **Huiyao Hao**: data curation, formal analysis, writing – review and editing. **Zhe Lv:** conceptualization, resources, supervision, writing – review and editing. **Yongzhou Song:** supervision, project administration, funding acquisition, writing – review and editing. **Yuzhong Wang** and **Tianlin Gao** have contributed equally to this work.

## Funding

This study was supported by the Key Research and Development Program of Hebei Province (22377752D), Medical Science Research Project of Hebei Province (20230486), Government‐Funded Clinical Excellent Talent Training Project of Hebei Province (ZF2024052), and Scientific Research Fund of The Second Hospital of Hebei Medical University, (2HC202210).

## Conflicts of Interest

The authors declare no conflicts of interest.

## Data Availability

The data that support the findings of this study are available from the corresponding authors upon reasonable request.
